# Nay to Prey: Challenging the View of Horses as a “Prey” Species

**DOI:** 10.3390/ani15050641

**Published:** 2025-02-22

**Authors:** Netzin G. Steklis, Mateo Peñaherrera-Aguirre, Horst Dieter Steklis

**Affiliations:** Human-Animal Interaction Research Initiative, School of Animal and Comparative Biomedical Sciences, University of Arizona, Tucson, AZ 85721, USA; nsteklis@arizona.edu (N.G.S.); steklis@arizona.edu (H.D.S.)

**Keywords:** prey hypothesis, mutualistic coevolution hypothesis, horse social cognition, equestrian research and training, human–animal interactions and relations

## Abstract

This paper challenges the prevalent characterization of domesticated horses as prey species that inherently view humans as predators. Drawing on evolutionary, ethological, and cognitive evidence, we propose the “mutualistic coevolution hypothesis”, which posits that horses and humans have evolved a partnership marked by cooperation rather than fear. We critically assess the “prey hypothesis”, emphasizing a predator–prey model, which dominates equine training and the literature, and we argue that it inadequately explains horses’ morphology, behaviors, and cognitive capacities. Comparative studies on horses’ socio-cognitive skills suggest that domestication has fostered emotional, behavioral, and cognitive adaptations supporting a human–horse bond. This review examines evidence from archaeological findings and experimental research on horses’ responsiveness to human gestures, emotions, and social cues, underscoring their complex cognition and capacity for collaboration. Furthermore, morphological and behavioral analyses reveal inconsistencies in using orbital orientation or predation-related traits as evidence for categorizing horses as prey species. By emphasizing the coevolutionary dynamics underlying human–horse interactions, we advocate for replacing traditional training models centered on fear and submission with approaches that leverage horses’ mutualistic and social nature. This perspective offers insights for enhancing horse welfare and improving human–equine relationships.

## Simple Summary

It is common among several horse trainers and researchers to claim that one of the goals of horse training is to overcome the animals’ natural apprehension towards humans. It is argued that this fear and anxiety originates from horses viewing humans as predators, a perspective known as the “prey hypothesis”. According to this perspective, horses view, in part, humans as predators due to our physical characteristics (for example, predators have frontal-facing eyes, whereas prey animals have their eyes on the lateral side of the skull). We reviewed the animal literature and concluded that the association between animals’ diet and different indicators of eye orientation is quite small, casting doubt on one of the premises of the “prey hypothesis”. Moreover, our analysis indicates that forward-facing eyes are associated with having a fruit- and leaf-based diet in primates. We also reviewed the scientific literature on horse’s mental abilities and emotional responses to humans. Horses exhibit diverse mental faculties that allow them to recognize human emotions and behaviors in social conditions, even when interacting with strangers. These findings strongly suggest that human–horse interactions are also cooperative, calling into question the precision and pertinence of the “prey hypothesis”.

## 1. Introduction

Among the various ecological interactions among biotic entities, predation remains a fundamental selective force for the evolution of an array of phenotypic traits. According to Taylor [1] the ethological literature provides several definitions for predation varying in their conceptual breadth. These include the following: (1) an organism kills and consumes another; (2) individuals of a particular species kill and eat individuals of a different species; (3) A process through which a population of a species obtains a benefit from a different population; and (4) It encompasses the flow of matter and energy between species. Consequently, if one takes Taylor’s first definition, a predator is an animal that kills prey for consumption.

The human characterization of domesticated horses as prey species and the consequent horses’ view of humans as predators is commonly expressed in equine publications and the horse training community. In this paper, we question this characterization of horses by critically examining the concept and criteria for classifying an animal as prey and reviewing relevant aspects of the evolution of human–horse symbiotic mutualism. We suggest that an evidence-based characterization of the domestic horse is not of an animal that is inherently fearful of humans but instead of an animal that has evolved cognitive and emotional traits suited for a mutualistic partnership with humans. We conclude with several implications of this different view of the horse for establishing the horse–human relationship.

### 1.1. The View of Horses as Prey and Humans as Predator

We begin with several examples from equine publications and the horse training community that explicitly characterize the horse as a prey species and its reactions to humans as a predator. In her exploration of how horses are represented in discourses of “natural horsemanship”, Birke [2] notes that those who look to build a partnership with the horse by relying on the principles of animal ethology see the horse as a prey animal “whose first reaction is flight” because it responds “to humans as predators”, or, more specifically, as “a predator on its back” (pp. 110–111). In Birke’s study, several equestrian researchers and trainers supportive of this view also argued that training outcomes depend on whether trainers recognize and act on the horse–human prey–predator interaction. Birke also describes exceptions to this view of horses in that some horse owners who, in seeking to establish a partnership, regard their horse “as almost human” rather than as a fear-dominated prey animal.

Further examples are provided in the published literature by notable horse experts, scientists, and trainers. In his article on natural horsemanship, Miller [3] observes that by using the body language of the horse, a prey species, the human (a predatory species) is quickly able to communicate with the horse to subordinate the horse and establish a bond with it (p. 1232). Likewise, Goodwin [4] remarks, “Defense strategies against human predators have not been reported in the literature, but predator defense strategies must be acknowledged and avoided in human-horse interactions” (p. 10). In discussing the potential problem of the suppression of pain by horses and other prey animals in developing a pain assessment tool, Dalla Costa and colleagues [5] note that horses may well suppress “the exhibition of obvious signs of pain in the presence of possible predators (i.e., humans) as is suggested with other prey species” (p. 1). The internationally well-recognized horse trainer and proponent of natural horsemanship, Parelli [6], has long and consistently similarly characterized the horse: “The main thing to understand is that horses are prey animals and people are predators” (p. 22). More recently, Parelli [7] emphasizes that the primary responsibility of a human toward his horse is “Don’t act like a predator”. A review of articles on the site Equisearch.com, prominently contributed to by trainer Emily Johnson [8], further echoes Parelli’s view of horses: “To achieve partnership, the horse must believe that although we look and smell like a predator, we are not going to act like a predator” and “Furthermore, any potential for a positive relationship between a horse and human depends on our understanding of them as a prey creature” [8].

Although we found that the view of the human–horse relationship primarily as one of predator–prey was widespread, there were some notable exceptions. For example, McGreevy and colleagues [9] proposed that horse handling and training are often classified either on the assumption that horses establish a generally cooperative interaction with their owners, handlers, or riders or that handling and training rely on human dominance and horse submission. Although this classification is adequate for horses acquainted with their trainers or owners, McGreevy et al. [9] recognize that it is insufficient when considering horses’ reactions to unfamiliar humans. According to the authors, handling and training protocols for naïve horses follow either a predator model, in which the horse behaves as a prey species in the presence of a potential predator, or the conspecific model, wherein the untrained horse interacts and displays a defensive–aggressive reaction similar to that of an agonistic interaction with a conspecific [9]. The authors note that one of the multiple limitations of the predator model is that it assumes horses rely on the cognitive heuristic that any dangerous stimulus is a predator and ought to be avoided. For the authors, this premise undermines the role of stimulus properties and perceptual processes in producing a behavioral reaction beyond a counter-predator strategy. This interpretation does not preclude horses from exhibiting anti-predator behaviors; however, it calls into question the preponderance of these perceptual, cognitive, and behavioral biases when considering the interactions between naïve horses and trainers.

### 1.2. The Prey Hypothesis and the Mutualistic Coevolution Hypothesis

As we have mentioned, the equestrian literature often discusses the “humans as predator” and “horses as prey” models. We will refer to this predator–prey model simply as the *prey hypothesis*. According to this hypothesis, horses are inclined to display fearful and anxious responses to humans, as in their evolutionary history, numerous predators hunted horses, including humans. This hypothesis asserts that several horses’ behavioral, cognitive, and emotional phenotypes have remained relatively unaltered over millennia, operating as fixed action patterns in response to negative stimuli. Through this lens, horses respond to a threat by first fleeing the area and then engaging in defensive agonism if the threat is unavoidable.

For example, Waran and collaborators [10] emphasized the effect of aversive stimuli on training prey animals like horses. According to the authors’ review of the training literature, fleeing is the default behavioral response of horses to aversive conditions. The authors warn against fear-inducing stimuli since the animals’ learning ability will be compromised, exposing handlers and trainers to hazardous conditions. In contrast, the authors recommend training procedures that calm horses and promote the animal’s and the trainer’s well-being. They provide descriptions of alternative horse training schools, such as the natural horsemanship school, which emphasizes a gentler approach to horse training. One of the objectives of this approach is for the animal to view the handler as an alpha in a dyadic association instead of a threat (i.e., predator). Accordingly, because prey species feel especially vulnerable when separated from their group, the alpha responds to a horse’s misbehavior by excluding the problematic individual from the group until the incorrect behavior disappears [10]. In other words, the trainer’s actions are designed to place the animal in an unpleasant condition until the animal directs its attention to the handler’s instructions. Per the authors, this interaction should be repeated so that the horse associates the trainer’s proximity with safety and comfort, establishing a hierarchical relationship between the handler and the horse.

In contrast to this prey hypothesis, we propose the human–horse *mutualistic coevolution hypothesis*. This hypothesis claims that the above-described emotional and behavioral responses to humans are not exclusively attributable to the horse’s ancestral predation; instead, horses display an array of cognitive abilities that allow them to interpret human emotions across various sensorial modalities accurately, an outcome of coevolutionary dynamics between humans and horses that superseded the ancestral ecological interactions among ancient hunters and equines. Consequently, when considering specific human–horse interactions, it is pertinent to consider the role of ontogenic, experiential, and individual differences, in addition to potentially robust fixed action patterns associated with anti-predator behaviors. Table 1 describes the various predictions associated with each hypothesis. The following sections review the comparative literature and provide evidence concerning the cognitive, behavioral, and affective substrates that support the prediction of the human–horse mutualistic coevolution hypothesis. The present study also explores the morphological predictions associated with the prey hypothesis using phylogenetic comparative methods.

### 1.3. Lack of Ecological Effects on Prey–Predator Ocular Adaptation

The orientation of animals’ orbits is often mentioned as evidence of differences in species’ diets and predatory behaviors. Thus, horses are characterized as prey species partly because their eye orbits face laterally with minimal visual field overlap, while the orbits of humans, like all predators, face straight forward, with high visual field overlap and depth perception. The argument goes that in horses and other prey species, the lateral orbit orientation, with a wider visual field, is an adaptation for predator detection, while the frontal orbit orientation in predators enhances the estimation of prey distance during a hunt. Several publications in the past have claimed that the evolution of frontal orbits was in part attributable to selective pressures favoring binocular vision (for a critique of these perspectives, see [11]). According to these studies, species featuring lateral orbits and, consequently, monocular vision were predicted to either lack or have limited stereopsis [11]. However, Timney and Keil [11] gathered perceptual data from domestic horses (binocular and monocular depth thresholds) and identified that the estimated binocular threshold was significantly better compared to the monocular threshold. The authors concluded that although horses have lateral orbits, they have significant stereopsis.

Moreover, the comparative data, do not support this long-held morphological difference between prey and predator. For example, Heesy [12] estimated the orbit orientation of more than one thousand specimens comprising 331 species within 16 extant mammalian orders and collected data on the taxa’s locomotor behavior and diet. The author conducted several multifactorial MANOVAs with activity pattern (e.g., diurnal, nocturnal), degree of faunivory (e.g., the proportion of animals in diet), substrate (e.g., arboreal, terrestrial), and interactions among the latter factors on orbit orientation. The model containing all mammalian species revealed that activity pattern explained the largest proportion of variance (*η*^2^ = 0.15), followed by substrate (*η*^2^ = 0.08; [12]). Notably, the species’ degree of faunivory accounted for a smaller proportion of variance (*η*^2^ = 0.05; [12]). The model also suggested that the interactions between activity, substrate, and degree of faunivory had a significant yet modest contribution to the model (*η*^2^ = range = 0.04 to 0.07; [12]). In short, these results show that diet on its own is insufficient to explain morphological differences in mammalian orbits.

Because most primates have frontally facing orbits and a large number of primate species could therefore bias the previous results, Heesy [12] re-examined the data by first restricting the data to all non-anthropoid species (i.e., subsample 1 excluding humans, apes, and monkeys) and all non-primate taxa (i.e., subsample 2 excluding humans, apes, monkeys, and prosimians). Per Heesy [12], a multifactorial MANOVA with subsample 1 revealed that orbit orientation was significantly predicted by species’ substrate (*η*^2^ = 0.11), followed by a faunivorous diet (*η*^2^ = 0.07) and activity pattern (*η*^2^ = 0.06). The interactions between activity pattern and faunivory (*η*^2^ = 0.06) and substrate and faunivory (*η*^2^ = 0.05) accounted for a small portion of the variance [12]. A similar pattern emerged in subsample 2, wherein orbit orientation was significantly predicted by substrate (*η*^2^ = 0.15), followed by the degree of faunivory (*η*^2^ = 0.08) and activity pattern (*η*^2^ = 0.07; [12]). The interaction between substrate and faunivory (*η*^2^ = 0.06) also explained little of the variance [12]. Although, in all models, faunivory reached statistical significance, this variable accounted for a negligible percentage of the orbit orientation variance across mammalian clades.

Additionally, in a recent phylogenetic comparative study on the macroevolution of orbits in carnivorans, Casares-Hidalgo and colleagues [13] measured the crania of nearly 200 carnivoran specimens corresponding to 107 species and 13 extant families. The authors also classified species according to their habitat, substrate use, and activity (i.e., visual strategy: scotopic, mesopic, or photopic). Like Heesy’s [12] statistical examination, Casares-Hidalgo and collaborators [13] also computed several MANOVAs to determine the influence of activity, habitat, and substrate on orbit orientation. Although the MANOVA detected a significant effect of activity, habitat, and substrate on orbit orientation, accounting for the shared phylogenetic history eliminated the influence of activity and habitat, while substrate significantly contributed to the model [13]. Consequently, Casares-Hidalgo and colleagues [13] concluded that most ecological factors have no contribution to the evolution of orbit orientation in carnivorans.

We further explored the effects of ecology on ocular morphology by gathering cross-species data from Heesy [14] on activity patterns (nocturnal, crepuscular, cathemeral, and diurnal), substrate use (terrestrial, variable, and arboreal), faunivorous diet (predominant, opportunist, omnivore, and none), orbit convergence (how much the orbits are angled towards the midline, indicating the degree of binocular vision), and orbit frontation (vertical orientation of the orbit relative to the skull, indicating how much the eye sockets point upwards or downwards; [15]). The final sample included 194 taxa of non-human animals, including Artiodactyla, Perissodactyla, Carnivora, and Primates. These data were used to examine two predictions associated with the prey hypothesis empirically. First, predators (e.g., faunivory) have greater orbit convergence and frontation. In contrast, “prey” taxa (e.g., non-faunivorous) feature limited orbit convergence and frontation.

Several Phylogenetic Generalized Least Squares Analyses (*caper* package, [16]) were subsequently conducted to determine the contribution of the aforementioned predictors after accounting for the data underlying phylogeny (all analyses were conducted in R v. 4.3.1). After controlling for the species’ phylogenetic history, we examined the influence of the activity pattern, substrate use, and diet indices on orbit convergence and frontation. The models revealed that orbit convergence was not significantly predicted by activity pattern, substrate use, or diet indices. Although orbit frontation was significantly predicted by substrate use, neither activity pattern nor diet had a significant influence on the model (see Supplementary Materials for further details).

It is possible, as an alternative hypothesis, that although orbit orientation is not significantly correlated with species ecology, pupil inclination is potentially linked with these environmental variables. For example, according to Hebel [17], horses feature a horizontal band that is associated with a considerable density of ganglion cells in the retina. To address this question we gathered data on pupil orientation, diel activity, and foraging mode from Banks et al. [18]. We restricted our examinations to mammals. The phylogenetic analysis revealed that although diel activity had a significant influence on the model, foraging mode did not influence pupil orientation (see Supplementary Materials for further details).

A derived prediction of the prey hypothesis argues that horses, as “prey” species, instinctively react fearfully to humans in part due to the frontal placement of humans’ orbits, which reflects our predatory past. However, this prediction ignores that most hominoids feature a considerable orbit convergence and frontation above and beyond the effect of diets. We empirically explored the latter prediction by examining the influence of diet on orbit convergence and frontation in a sample of non-human primates. Lower orbit convergence and frontation are expected to be associated with folivorous/frugivorous diets. Consequently, if the statistical analyses either fail to detect a significant effect of diet on orbit orientation and frontation or reveal effects running in the opposite direction, such results would falsify the prediction that diet is a major predictor of orbit morphology in primates and further call into question the assertion that humans feature frontal orbits due to their predatory past. To test the latter prediction, we conducted a preliminary comparative phylogenetic analysis of 68 species of non-human primates.

**Figure 1:**
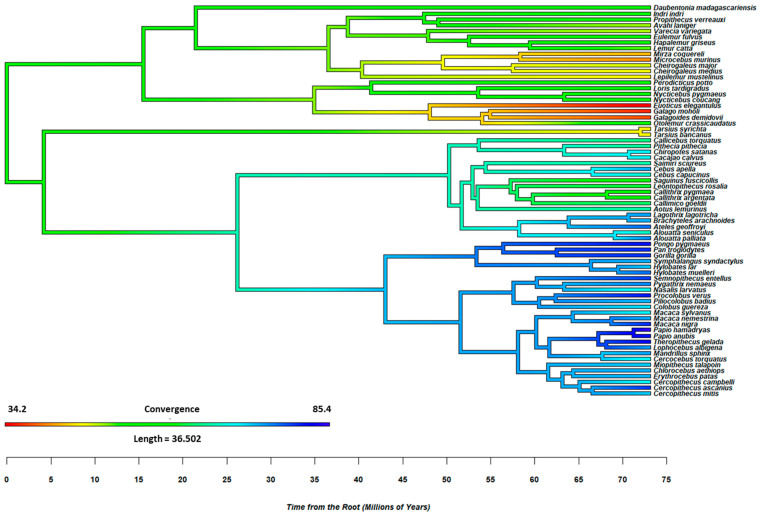
Ancestral character reconstruction of orbit convergence in a sample of non-human primates.

**Figure 2:**
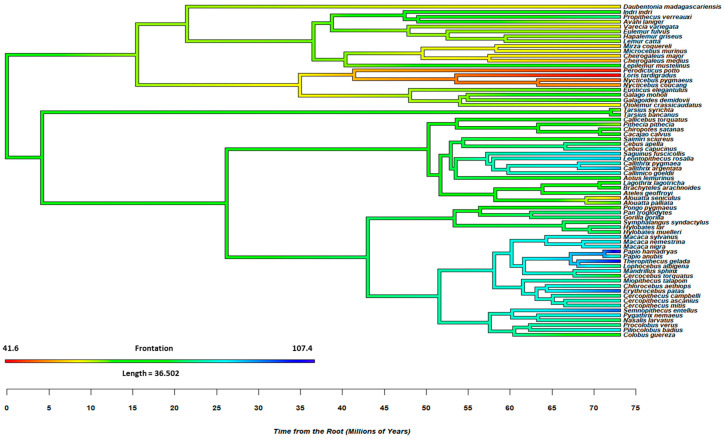
Ancestral character reconstruction of orbit frontation in a sample of non-human primates.

The models considered the influence of diet (frugivore, folivore, and omnivore; [19]) on orbit orientation, which is operationalized by convergence and frontation. The analyses controlled for phylogeny and included as predictors two orthogonal contrasts (C1: omnivores vs. frugivores/folivores; C2: frugivores vs. folivores). Figure 1 and Figure 2 provide further details on the evolutionary conservation of orbit convergence and frontation among non-human primates. The results of phylogenetic examinations suggested that orbit orientation is phylogenetically conserved, with omnivorous species evidencing lower convergence and frontation. In other words, omnivorous non-human primate species have *less* forward-facing eyes than frugivore/folivore species (see Supplementary Materials for further details).

Additional studies have also considered the role of primate neuroanatomy on binocularity, reporting sizeable effects. For example, Barton [20] employed phylogenetic comparative methods to estimate the correlation between orbital convergence and various neuroanatomical volume indicators in non-human primates. The relative size of the lateral geniculate nucleus (LGN) and the overall volume of the neocortex, respectively, explained more than 30% of the variance [20]. The volume of area V1 accounted for a smaller percentage of variance (23%; [20]). Subclade analyses revealed that these effects increased in magnitude within diurnal anthropoid primates, with the relative size of the LGN and neocortex, respectively, accounting for 64% and 48% of the variance [20]. Similarly, area V1 explained 30% of the variance [20]. Although additional studies are required to determine the proportion of unique variance explained by neuroanatomy relative to diet, the magnitude of the latter effects (after controlling for phylogenetic and allometric effects) suggest that, at least in primates, diurnal neuroanatomy is an essential correlate of orbit orientation.

Finally, within the framework of the prey hypothesis, humans as predators should exhibit greater orbit convergence and frontation (i.e., greater binocular stereopsis). However, comparative examinations of orbit morphology among our closest relatives, the hominoid primates, strongly indicate that humans and gibbons, compared to chimpanzees, gorillas, and orangutans, have less convergent orbits [21]. Moreover, human orbit morphology gives us superior lateral vision relative to that of other primates [21]. Similarly, a horse’s orbit morphology falsifies the notion that orbit convergence is essential for binocular stereopsis, as Read [22] acknowledges: “Virtually all animals have some binocular overlap, however, and the number of animals found to have some form of stereopsis is increasing. Yet, our thinking about stereopsis is still highly anthropocentric. Even in papers on non-primate vision, it is common to find misconceptions such as the idea that stereopsis requires high visual acuity, frontal eyes, or a large binocular overlap (the horse disproves all three)” (p. 390). These studies show that orbital orientation is not a reliable indicator of predator or prey niches.

### 1.4. Horse Anti-Predator Behaviors

As we have shown earlier, horses are described as responding to humans as prey to predators. Much research, however, indicates that horses’ responses to predators vary between horse breeds. Janczarek and colleagues [23] reproduced the vocalizations of several carnivoran species (grey wolf, golden jackal, and Arabian leopard) to Arabian and Konik polski horses. The authors gathered data on the horses’ time grazing, remaining still, staying alert, and physical proximity to the speaker (operationalized as the number of steps). Their analyses revealed significant behavioral differences between horse breeds. For example, Arabian horses displayed greater arousal levels in response to the Arabian leopard’s recordings than Konik polski horses, including a shorter duration of grazing, remaining still and walking, and spending more time alert and trotting. Moreover, Koniks featured a higher frequency of relaxed responses such as grazing, remaining still, and walking [23].

In contrast, Konik polski horses exhibited more distress when exposed to the wolf’s recordings (more prolonged duration of alert state and more trotting; [23]). The authors also examined the animals’ social behavior in response to the carnivorans’ vocalizations. The statistical models revealed that the herds of Konik polski horses displayed a tighter formation and avoided the loudspeaker when presented with the wolf’s recording [23]. The Arabian horses approached the loudspeaker playing the leopards’ vocalizations, following a horizontal linear formation (the animals advanced next to each other; [23]).

Rather than suggesting the presence of general anti-predator strategies in horses, the results strongly indicate that horses’ individual and social responses vary depending on the predator species and the horses’ breeds.

The prey hypothesis stipulates that horses have retained their innate fear responses to humans and other predators. However, an experimental study of behavioral and physiological (i.e., heart rate) reactions in horses to various predator odors [24] casts doubt on an automatic fear response to predators. The authors tested the common belief among riders that horses are innately frightened by predator odors by gauging horses’ reactions to urine from wolves and lions, wolf fur odor, and slaughtered horse blood. Their results showed that while exposure to various predator odors led to increased vigilance, the horses showed no evidence of fear (e.g., heart rate was not elevated). Heart rate increased only when an additional “fear-eliciting stimulus” (i.e., a sudden noise) was paired with the predator odor. Based on these results, the authors suggest that horses retain some innate responses to predators but that “the selection pressure for predator recognition has inevitably been relaxed through domestication” (p. 142). Together, these studies suggest that today’s horses do not have a universal, innate response to predators, likely including humans.

### 1.5. Homo–Equus Mutualistic Coevolution: From the Eneolithic to the Bronze Age

As an elaboration of the *human–horse mutualistic hypothesis*, the following section further reviews the current zooarchaeological literature to emphasize the extended coevolutionary process between humans and horses, as manifested by the non-predatory association between humans and horses established since the Bronze Age. This review will begin by describing the results of proteomic and morphological studies in Eastern European archaeological sites.

Outram and colleagues [25] provide several lines of evidence concerning early human–horse associations beyond predation. The authors examined the remains of horses recovered from Eneolithic Botai culture sites in Kazakhstan (~3500 BC). They determined that the metacarpals of Botai horses shared several morphological features with subsequent Bronze Age horses instead of wild horses extracted from Paleolithic strata at the exact same location. The authors also argued that Botai horses displayed pathological signs associated with frequent bridle usage. Moreover, the analysis of organic material found in Botai ceramics suggested that the pottery contained mares’ milk and fat residue [25]. Although zooarchaeologists continue to debate whether the Botai rode horses, the latter evidence indicates this culture co-existed in close physical proximity to horses, suggesting high levels of interdependence. Similarly, Wilkin and collaborators [26] obtained samples from over 30 Bronze Age individuals recovered from several archaeological sites between the Volga and the Ural mountains. The investigators classified their samples according to their chronology, with the first group ranging from 3300–2500 BC (Early Bronze Age) to 2500–1700 BC (Middle-to-Late Bronze Age). Whereas the remains of the first chronological group corresponded to the Yamnaya culture, individuals from the second group were affiliated with the Sintashta culture. Analyses of the human dental calculus indicated significant differences in dairying practices in the Volga–Ural steppes [26]. Consequently, the latter evidence supports the presence of non-predatory associations between horses and humans since the Bronze Age.

In addition to molecular data, the archaeological record of the relative number of domesticated animal remains provides further evidence that Bronze Age humans did not primarily hunt and consume horses, suggesting that they moved toward establishing coevolutionary non-predatory interactions between humans and horses. In his review of the zooarchaeological literature, Anthony [27] identified variations in the percentage of domesticated animal remains in Early Bronze Age settlements and graves. For example, Yamnaya graves, located in the Don–Volga steppe, featured a large percentage of sheep and goat remains (65%), followed by cattle (15%), horses (8%), and dogs (5%; [27]). In Mikhailovka II-III, a Yamnaya settlement, cattle comprised most of the animal bones (59%), followed by sheep and goats (29%), horses (11%), pigs (9%), and dogs (7%; [27]). Lastly, a settlement located in Repin featured a larger percentage of horses (55%), followed by cattle (18%), sheep and goats (9%), and pigs (9%; [27]). Anthony [27] described the aforementioned differences in animal bone remains as partly attributable to differences in mobility and subsistence economy between Yamnaya communities. Consequently, cattle remains are more frequently found in settled communities, while mobile groups feature a greater percentage of goats, sheep, and horses [27]. By the Mid–Late Bronze Age, horses were no longer the primary hunted animal [27].

### 1.6. Previous Reviews of Human–Horse Relationships

The present paper is not the first publication that has reviewed the nature of horse–human relationships. For example, Hausberger and collaborators [28] emphasized that learning rules and schedules are not often mentioned in the equestrian training literature. Moreover, according to the authors, caretakers and trainers frequently employ procedures wherein the animal learns to avoid experiencing an unpleasant stimulus. Hausberger and colleagues [28] also argued that horse training suffers from excessive punishment to reduce the frequency with which a specific behavior is performed. According to the authors, the overuse of punishment increases the likelihood of the following: (1) horses experiencing motivational conflict due to a lack of behavioral control in a particular situation; (2) the animals experiencing high levels of stress; and (3) the performance of agonistic behaviors such as kicking, biting, and rearing, among others. Although the authors caution against excessive punishment, their review does not endorse a reward-based system. For example, physical contact with the animal (e.g., patting) may be ineffective [28]. Similarly, a reward given at an inadequate time interval could increase the frequency of undesirable behaviors [28]. Consequently, Hausberger and collaborators [28] recommend including learning rules in equestrian training, promoting harmonious horse–human relationships based on mutual positive experiences.

Similarly, Fureix and colleagues [29] examined the cognitive ability of horses to generate memories of specific people based on past interactions with humans. According to the authors, the study’s results suggested that horses’ behavioral responses to people are partially attributable to the nature of previous interactions. The quality of social relationships with humans during horses’ development has also been found to contribute to cognitive processes. For example, Sankey and collaborators [30] examined the memory of more than 20 young domesticated horses when recalling information about humans after experiencing varying temporal separations (six and eight months). The statistical models indicated that individuals exposed to positive reinforcement performed better in learning- and memory-related tasks than individuals in the control condition [30]. The authors also compared the frequency of positive (sniffing and licking) and negative (biting and kicking) behaviors directed at the trainer during the positive reinforcement and control conditions. The statistical models revealed that sniffing and licking occurred more frequently under positive reinforcement compared to in the control condition [30]. Moreover, the analyses suggested that biting and kicking behaviors were less frequent in the positive reinforcement conditions relative to in the control condition [30]. The latter studies and reviews offer further insight into the influence of learning rules and the quality of human–horse relationships when considering horses’ social cognition and behaviors.

### 1.7. Horse Attention to Human Gestures and Social Information

A review of horse cognition, especially its attunement to human gestures and other communicative expressions, casts doubt on the notion that horses see us as predators and fear us (i.e., the prey hypothesis). Instead, these studies show that horses are equipped cognitively and motivated to see humans as partners or collaborators, reflective of an evolved mutualism. For example, Proops and McComb [31] examined horses’ cognitive ability to identify the direction and object of human attention. The authors gathered behavioral data from horses exposed to inattentive or attentive people with food rewards. The authors allowed subjects to choose between two experimenters under four conditions. (1) Body orientation: one of the experimenters’ bodies faces toward the subject; the body of the remaining experimenter faces away from the subject. (2) Head orientation: both experimenters position their bodies towards the horse; however, the head of one of the experimenters faces away from the subject. (3) Eyes: both experimenters direct their bodies and heads toward the animal; one has their eyes open, and the other has them closed. (4) Combined: the attentive researcher’s face was directed toward the subject but their body was oriented in the opposite direction, but the inattentive researcher’s face was directed away from the subject, and their body was oriented toward the subject. Proops and McComb [31] also evaluated two experimental variations in the latter conditions. In the first one, the person’s body faced away from the horse, but their head was oriented toward the animal (attentive variation). In the second variation, the person’s body faced toward the horse, but their head was oriented away from the animal (inattentive variation; [31]). The subjects’ percentage of correct responses was statistically significant for body orientation, face orientation, and eyes open or closed [31]. No significant differences were detected for the combined setting [31]. The study also revealed that female horses performed better compared to males, while age was not significantly associated with the subjects’ performance. These results show that horses can correctly infer a human’s attentional focus.

Although the latter examination did not detect an age effect, more recent studies have provided further insight into the ontogeny of socio-behavioral and cognitive traits in horses. For example, Proops and colleagues [32] evaluated the behavioral performance of young subjects relative to that of adults. Their analyses indicated that individuals below three years of age could accurately interpret the person’s body orientation, but they were incapable of understanding more subtle cues such as head orientation or eye direction compared to adult subjects. In terms of the object choice tasks, the authors discovered that although younglings accurately interpreted sustained distal pointing, they did not understand cue tapping, body orientation cues, or gaze alteration cues [32]. Moreover, few younglings approached the person’s extended arm prior to exploring the container, whereas in adults, more than half of the animals performed the latter behavior [32].

Horses also can accurately interpret human manual gestures, as demonstrated in a study of domestic horses by Maros et al. [33]. Several experimental tasks required the horses to accurately interpret the researchers’ gestures, including the following: (1) The experimenter pointed at a baited bucket for 1 s using her index finger while looking at the animal [33]. The experimenter then retracted her arm (i.e., “distal momentary pointing”). (2) The experimenter pointed at the bucket using her extended arm and index finger while looking at the animal (“distal dynamic sustained pointing”; [33]). (3) This was similar to condition 2, except the researcher sat next to two buckets at a distance of 10 cm from her index finger while looking at the horse and pointing at the baited bucket and then retracted her arm (“proximal momentary pointing”; [33]). (4) This was similar to condition 3, except the experimenter looked at the baited bucket (proximal dynamic-sustained and gazing tasks; [33]). The horses’ performances were statistically significant for the distal dynamic-sustained, proximal momentary, proximal dynamic-sustained and gazing tasks but not for the distal momentary condition [33].

A more recent study by Trösch and collaborators [34] provides evidence that horses attend to and use information acquired by an unfamiliar human. Nineteen individuals were used in an experimental protocol divided into three phases. In phase one, a research assistant entered the experimental arena with the horse facing an empty bucket [34]. Two experimenters unfamiliar with the horse stood next to the bucket, with the first individual facing away and the second facing toward the container [34]. In phase two, a second research assistant entered the room and placed a food item inside the bucket [34]. In phase three, the two experimenters turned and faced the horse [34]. In the control condition, the research assistant presented the food reward directly to the horse before putting it inside the container [34]. The statistical analysis revealed that horses observed the individual who had a direct line of sight to the bucket longer than those who faced away from the container [34]. No significant differences were detected during the control condition [34]. The horses also physically interacted more frequently with the individual who observed the food item placed inside the bucket compared to with the person who had no line of sight to the container [34]. Although it is controversial to argue that horses have a theory of mind (the ability to attribute mental states to others) instead of a theory of behavior (drawing inferences based on others’ behavioral sequences), these results suggest that horses have the cognitive faculty of gathering relevant social information from unfamiliar humans.

Individual recognition is an essential component of daily social interactions and, as such, can be seen as essential to the establishment of a mutualistic relationship. Proops, McComb, and Reby [35] explored whether domesticated horses can identify a herd member across perceptual modalities. The authors recorded long-distance vocalizations of isolated horses from the same herd. They then exposed the subject to a herd-mate, who eventually was led out of view. Following a 10 s interval, the experimenters played incongruent recordings (the vocalization did not match the herd-mate out of view) or congruent (the vocalization matched the herd-mate out of view) and collected the behavioral responses from 24 individuals. The results indicated that subjects featured shorter reaction times and faced the speaker’s direction more frequently and for a longer time in the incongruent condition compared to in the congruent one [35]. Additionally, the nature of the relationship between herd-mates did not contribute to the subject’s reactions (in either congruent or incongruent conditions).

Consistent with the human–horse mutualistic coevolution hypothesis, horses can also recognize and discriminate between humans. Lansade and collaborators [36] introduced horses to four unfamiliar human faces (presented as colored portraits). The authors then presented the animals with four modifications to the facial portraits, including altered hairstyles, concealed eyes, black and white colors, and profile views. The testing phase entailed introducing a novel face next to a recurrent face in any of the aforementioned variants [36]. The subjects performed above chance across all experimental conditions when the recurrent face was present in any version [36]. In contrast, during a control condition (the horse was introduced to two novel faces), the subjects did not perform above chance [36]. These results show that in horses, as in humans, facial recognition is a holistic perceptual–cognitive process wherein multiple perceptual cues are integrated to infer the identity of a heterospecific.

The horse–human mutualistic coevolution hypothesis also predicts that horses can recognize human emotional expressions. Evidence indicates that domesticated horses can distinguish between human facial expressions of emotion. Smith and colleagues [37] introduced horses to color photographs featuring happy and angry human facial expressions. Based on previous evidence for a left-gaze bias in dogs for human angry facial expressions, Smith and collaborators [37] established a laterality index to determine the amount of time horses spent observing each photograph (left gaze, right gaze, or straight). They found that a larger number of horses used their left eye when viewing angry facial expressions for the first time. Similarly, individuals showed a left-gaze bias for time spent observing the photographs [37]. Although the analyses did not reveal a significant difference in avoidance, approach, or duration between emotional stimuli, there was a positive and significant correlation between avoidance duration and the time of left-gaze bias [37]. These results strongly indicate that left-gaze bias, as a proxy for emotional distress to negative stimuli, is not exclusive of traditional animal companions, such as dogs, but is also present in domestic horses. Additional studies are required to determine whether this phenomenon is a convergent evolutionary process due to the mutualistic connection of humans with dogs and horses.

The various cognitive capacities discussed above may not have evolved during the course of horse domestication as part of horse–human mutualism but instead reflect ancestral cognitive adaptations to herd social life. Nevertheless, these socio-cognitive skills surely facilitated the development of horse–human mutualism. The complexity of the latter cognitive abilities, such as the cross-modal recognition of human emotions (even in strangers), requires present and direct contact between humans and horses. Thus, the ancestral ecological interactions between human hunters and ancient horses are unlikely to provide the most parsimonious evolutionary explanation of fear and anxiety responses in domesticated horses.

Recent studies have examined potential factors contributing to horses’ understanding of human communicational cues. Liehrmann and collaborators [38] evaluated domestic horses in a two-way choice experiment. The authors considered the influence of social factors such as the length of the relationship between each horse and their corresponding caretaker and whether the animals lived in groups, dyads, or alone. Liehrmann and colleagues [38] also explored the effects of living conditions, including whether the horses were housed in stalls or paddocks or spent most or all of their time in fields and pastures. The statistical analyses revealed that animals kept in groups outperformed those living in dyads or alone [38]. The authors also reported that living conditions altered the subjects’ scores, wherein individuals living outside in fields or pastures featured better performances than those living in paddocks. Additionally, younger animals performed worse compared to more mature individuals [38]. These results strongly suggest that maturational effects, in conjunction with social and living conditions, influence horses’ cognitive capacity to understand human communication cues, providing further evidence for the coevolution mutualistic hypotheses.

Under the prey hypothesis, feral and captive horses (including young horses) would be expected to exhibit similar fearful responses to humans. However, the evidence does not support this position. For example, Górecka-Bruzda and colleagues [39] conducted a longitudinal study to determine the degree to which Konik polski horses differed in their response to humans depending on whether the animals were born in captivity (stable born) or a semi-feral herd (forest-born). The authors gathered ethological data from forest-born and stable-born animals at various developmental stages (6 to 18 months). According to the authors, more stable-born animals walked toward the experimenter and were more receptive to touch than forest-born individuals. In contrast, forest-born horses displayed a greater latency and flight initiation distance than stable-born individuals [39]. Moreover, the authors discovered significant physiological differences between foal categories. For example, forest-born foals featured greater mean heart rate and heart rate variability than stable-born foals [39]. Although the authors did not detect any significant differences in the animals’ overall fearfulness to startling stimuli, older stable-born animals regulated their behavior and emotions more effectively than forest-born subjects. These results suggest that rearing and social conditions influence horse–human interactions and are not as predicted by the prey hypothesis.

### 1.8. Additional Comparative Evidence Countering the Prey Hypothesis

Phylogenetic comparative research also questions the notion of horses as a prey species. For example, previous macroevolutionary examinations suggest that ungulate species featuring fast running speed are more susceptible to suffering from capture myopathy (a physiological syndrome evidencing severe damage to cardiac and circulatory tissues due to high emotional distress such as a chase or capture, [40]). Although several perissodactyls are susceptible to capture myopathy, neither Przewalski nor domesticated horses suffer from this physiological condition [41]. According to Steklis and colleagues [41], of the several extant equine species, only zebras display capture myopathy. A review of the paleoecological literature led the authors to conclude that the number of medium and large carnivoran predators (felines, canids, hyenas, and ursids) declined significantly in the Palearctic and Saharo-Arabian regions, relative to the Afrotropic between the Pleistocene and the Holocene. The percentage of predator loss was negatively correlated with the number of equine species featuring capture myopathy [41]. Consequently, unlike other equine taxa exposed to high predation pressures, Palearctic equines, such as domestic horses, became less vulnerable to cardiac over-exertion.

It could be argued that horses’ negative behavior toward people riding them is in part attributable to riding resembling a predator pouncing on the animal. However, the ecological literature on the type of carnivorans preying on horses falsifies this hypothesis. Boyce and McLoughlin [42] conducted a literature review on the main predators of feral horses across contemporary nations. The authors identified considerable variation across species and nations in the carnivorans’ percentage of feral horse meat in their diet (FHD). For Europe, the authors reported that: (1) in Spain, the FHD for wolves ranged from 34% to 37% [42]; (2) in Portugal, the FHD for wolves was 41% [42]; and (3) in Italy, the FHD for wolves ranged from 1% to 7% [42]. Further east, studies with Asian populations evidence a similar pattern, wherein: (1) in Mongolia, the FHD of wolves and snow leopards was 32% and 12%, respectively [42]; and (2) in Nepal, the FHD of snow leopards and wolves was 11% and 9%, respectively [42]. Evidence concerning the killing of feral horses in Oceania is restricted to Australia, with dingoes featuring FHD values between 3% and 5% [42]. These results show that wolves are the predominant predators of feral horses in Eurasia, followed by snow leopards.

In contrast, in the Americas, felids, rather than canids, mostly prey on feral horses. Significant differences have also been reported in the consumption of horse meat across North American regions. Whereas the FHD values for pumas in California and Nevada range from 45% to 77%, in Alberta, the same species features lower FHD values that range from 10% to 13% [42]. Lastly, relatively few studies have been published on this subject in South America. According to the author, in Brazil, the puma FHD value was 51% [42]. Additional studies are required to determine if this pattern extends to other parts of South America. These results indicate that felid predation occurs more frequently in the Americas than in Eurasia or Oceania; however, even in the New World, the FHD values for felids vary across regions. The latter study indicates that locations around the world differ in the percentage of predatory attacks, calling into question the hypothesis that horses reject riders because the animal views riding as equal to a predator attack.

Additional cross-equid studies have also examined whether horses and zebras differ in their avoidant behaviors. Bubaker and Coss [43] contrasted the start distance (the starting point between the human researcher and the location of the animal), the alert distance (the point at which the animal detects the person), and the flight initiation distance (the point at which the animal flees the location; a buffer distance was also measured as the physical span between the alert point and the flight point) of free-ranging plains zebras and US feral horses. The authors examined the interaction between species type and individuals who experienced low or high exposure to humans. The analyses revealed a significant interaction between exposure and species, with high-exposure horses featuring the lowest values [43]. Alert distances were more extensive for low-exposure zebras and feral horses than for high-exposure equids [43]. Lastly, the models identified a significant main effect of exposure on buffer distances [43], with alert and buffer distances positively correlated. Overall, these results indicate that rather than operating as an undifferentiated behavioral sequence, the behavioral responses of wild equids vary as a function of exposure, species, and various types of distances. Additional studies are required to compare flight initiation distances between free-ranging and captive zebra species with domesticated species.

The prey hypothesis ignores that predators are also preyed on by other species [44]. Moreover, the socioecological effects on mammalian anti-predator vigilance are inconsistent with the notion of greater vigilance in folivorous relative to carnivorous species. A prediction derived from the prey hypothesis is that if the likelihood of capture by a predator increases in smaller groups, vigilance is expected to be negatively associated with group size in mammals, especially in non-carnivorous animals. Beauchamp and collaborators [45] performed a systematic review and meta-analyses to determine the overall effect of group size on vigilance in mammals. The authors collected more than 290 effect sizes corresponding to 97 mammalian species within 10 orders. Analyses indicated that having a carnivorous or folivorous diet did not significantly predict the correlation between group size and vigilance [45]. Similarly, the overall correlation between group size and vigilance did not differ between grazers and non-grazers [45]. The authors also considered whether the latter correlation remained significant when comparing the predation risk; no significant effect was detected. These results strongly suggest that the species’ diet or predation risk is statistically irrelevant to our understanding of a species’ vigilance.

Species descended from larger carnivorans, such as domestic dogs, are not exempt from predation. For example, Butler and colleagues [46] conducted a review to determine the frequency of wild carnivorans preying on domestic dogs around the globe. Although the authors identified biogeographical differences in the frequency of carnivoran predation, their analyses suggested that grey wolves, followed by leopards, pumas, coyotes, and hyenas, more frequently attacked and preyed on dogs. Butler and colleagues [46] also concluded that other carnivoran taxa killed domestic dogs at a lower frequency. Other species included tigers, lions, dingoes, striped hyenas, jaguars, black-backed jackals, polar bears, and Asiatic black bears. Butler and collaborators’ [46] review also revealed that several non-carnivoran species kill domestic dogs, such as Chacma baboons, cassowaries, saltwater crocodiles, and freshwater crocodiles. Their results are consistent with the aforementioned studies concerning the risk and rate of predation experienced by carnivorans. Consequently, the distinction between predator and prey based exclusively on operationalizing the presumed foraging mode ignores the complex biotic interactions (e.g., trophic chains) among taxa within their community ecology. Moreover, such ecological interactions extend to environments modified via human cultural niche construction, wherein in some environments, domestic species (e.g., dogs) are vulnerable to conflict with or predation from wild fauna.

Additionally, interspecific amicable or tolerant relationships have also been reported among animals above and beyond traditional competitive or predatory interactions. For example, Dagg [47] assembled numerous instances of cross-species “friendships”, including the following: (1) a crow and a moose; (2) multiple goats and a baboon; (3) a cat and a groundhog; (4) several cardinals and robins; (5) a chimpanzee and a baboon; (6) an elephant and a dog; (7) a coyote and a badger; and (8) several goats and a wolf. Moreover, several studies have examined the diversity of interactions between cats and dogs. For example, Menchetti and colleagues [48] collected questionnaire data from participants who owned dogs and cats. The authors concluded that dogs and cats prefer to eat separately. A larger percentage of dogs groomed cats than vice versa [48]. In turn, a greater number of cats ignored their canine counterparts [48]. The authors say more than 60% of dogs and cats play and sleep together. Consistent with folk-based descriptions, agonistic interactions between dogs and cats also occur. For example, Shamir and colleagues [49] examined the type of dog bite injury in cats and dogs. The authors concluded that out of more than 196 cases of dog bites, 6% were to cats and 94% to dogs. Although additional studies are required to determine the frequency to which these heterospecific interactions emerge across animal clades, the latter evidence suggests that the outcome of biotic interactions occasionally does not adhere to traditional ecological classifications such as predation or competition.

The prey hypothesis further presumes that animals classified as predators will not respond to their removal from the group or their introduction to an unfamiliar setting, with limited emotional arousal. Although individual and breed differences and attachment styles influence the behavioral responses of dogs to Ainsworth’s Strange Situation experiments, the ethological literature shows that dogs experience emotional stress when interacting with a stranger in a novel scenario [50]. A stress response is not unexpected for a social animal, as sociality provides fitness benefits to predatory animals, above and beyond the spoils of cooperative hunting, including defense from conspecifics and heterospecifics. Even though it is not uncommon for trainers in the canine literature to analogize the social structure of domestic dogs to that of grey wolves, socioecological examinations strongly suggest that the symbiotic association between dogs and humans significantly altered the ancestral canine social structure [51]. The social group of domestic dogs comprises hetero- (humans and other pets) and conspecifics (other dogs). Removing a domestic dog from its social group may induce alertness or fear due to the animals’ exposure to potential threats. Thus, the negative response of either dogs or horses is not attributable to their alleged predatory or prey nature but to their evolutionary and ecological reliance on their social groups. Additional studies are required to determine the influence of attachment styles on horses’ behavioral response to the Strange Situation.

The prey hypothesis implies that the horse’s current fear was shaped primarily in response to predators. However, in addition to predation, we must consider the conspecific lethal aggression rate as another danger source to mammalian species. Gomez and collaborators [52] assembled a database on lethal aggression across mammals, consisting of information from the behavioral ecological literature on mammalian adulticide for more than 350 species in 29 orders. For the present paper, we estimated the percentage of adulticide per mammalian order based on the supplementary data found alongside Gomez and colleagues’ publications [52]. Our brief analyses suggest that adulticide was present in 54.3% of Artiodactyls, 44.3% of carnivorans, and 33.3% of Perissodactyls. Gomez and colleagues [52] computed several phylogenetic logistic regressions to determine the influence of body size, sexual dimorphism, polygyny, group size, population density, infanticide, carnivory, and the presence of intrasexual armaments on male and female adulticide. Concerning male adulticide, the authors found that body size sexual dimorphism, infanticide, and intrasexual armaments positively predicted male adulticide. In turn, population density had a negative significant effect on the model, while infanticide had a positive and significant influence on female adulticide [52]. In both analyses, a carnivorous diet did not significantly influence either male or female adulticide [52]. These results strongly suggest that the risk of suffering conspecific lethal aggression in adults is not due to relying on a specific diet but instead is predominantly attributable to intrasexual competition and sexual coercion. Although the analyses did not disaggregate the data based on within-group compared to between-group conflict, they provide further insight into the predictors of lethal interactions among mammals. As adulticide is quite prevalent across mammalian orders, future studies are required to examine whether the distress experienced by some mammalian species when removed from their social group is in part predicted by the rate of conspecific aggression perpetrated by outgroup rivals (during territorial incursions) or ingroup members attacking the target when it is isolated from kin and allies.

### 1.9. The Influence of Domestication on Animal Cognition

Jardat and Lansade’s [53] review of the socio-cognitive abilities of domestic animals indicates considerable overlap between dogs and horses in the following: (1) perception of human faces based on cognitive discrimination, recognition, and cross-modal representation; (2) perception of human emotions based on cognitive discrimination, cross-modal representation, and physiological and behavioral responses; (3) interpretation of human intentionality, such as sensitivity to apparent cues and attentional states; (4) communication including recognizing human cues, referential communication, and performing communication attempts; and (5) social learning, such as local enhancement and social referencing. Using the data summarized in Jardat and Lansade [53], we computed the proportion of cognitive abilities shared between horses and other alleged prey species and two so-called predator species: dogs and cats. Horses and dogs share thirteen cognitive abilities manifested during interactions with humans. Similarly, horses and cats have ten abilities in common. Lastly, dogs and cats share eleven abilities. According to Jardat and Lansade [53], few cognitive studies have been conducted with other domesticated animals, such as sheep, pigs, goats, and cattle. Although subsequent examinations should consider whether these domesticated species have multiple socio-cognitive abilities in common with dogs, cats, and horses, the present results strongly suggest that these cognitive capacities are not restricted to a particular taxon as a function of its diet.

Additional publications have examined whether domestic and wild species differ in cognitive abilities. For example, Ferreira and collaborators [54] determined that wolves outperformed dogs in problem-solving and spatial orientation, quantity discrimination, use of physical cues, discrimination and reversal learning, and social learning from conspecifics. Dogs and wolves exhibit similar performances in tasks assessing object permanence, means-end connections, memory, working memory, cooperating with conspecifics, cooperation with humans, and local enhancement [54]. In contrast, dogs outperformed wolves in tasks measuring sensitivity to human cues [54]. These results suggest that the cognitive similarities between wolves and dogs, especially concerning social cognition towards conspecifics, are attributable to their shared phylogenetic history.

In contrast, the noticeable variation in performance in socio-cognitive tasks during animal–human interactions also suggests that these phenotypic differences are partly attributable to coevolutionary dynamics between domestic dogs and humans. Although fewer comparative studies have been conducted with other taxa, examinations between wild and domesticated mustelids indicate that domestic ferrets outperform their wild counterparts in tasks assessing the use of human cues [54]. Ferreira and colleagues’ review [54] provides further insight into the significant heterogeneity in cognitive performance between domestic animals and the corresponding wild lineages. As the nature and quality of the relationships between humans and domestic animals differ depending on the taxa [55,56], it is expected that domestic animals who are not used for consumption and instead predominantly interact with humans as either companion or as work animals should be cognitively and affectively more attuned to human emotions and behaviors. Hence, the present paper does not argue that domestication per se produced horses’ attunement to human emotions and behaviors; instead, it was the coevolution between humans and horses through a specific domestication pathway based on a non-predatory interaction that favored the rise and persistence of the traits discussed in this paper.

It is worth noting that human–animal coevolutionary dynamics are not the only source from which animals understand human pointing. Miklosi and Soproni [57] surveyed the literature on this subject and classified studies based on their temporal dimension (static v dynamic) and their orientation (based on body signaling, proximal pointing, distal pointing, cross-pointing, asymmetric pointing, and elbow cross-pointing). According to the authors, dogs, cats, dolphins, and seals can understand dynamic and momentary distal pointing. Although dogs and chimpanzees correctly understand body signaling, only dogs and seals adequately understand asymmetrical pointing [57]. Their review also suggested that even though wolves correctly followed proximal dynamic pointing, as do dogs, cats, and capuchin monkeys, their percentage of correct responses was considerably lower. Moreover, wolves underperformed dogs and cats in terms of distal dynamic pointing [57]. Future studies are needed to determine the influence of socioecological factors and neuroanatomical volume indicators in the evolution of these socio-cognitive abilities.

### 1.10. Further Considerations of Human–Horse Interactions

The latter review of fundamental comparative studies is predominantly restricted to developed nations. Although additional studies are required to determine their generalizability to other locations, current reviews and meta-analyses on horse welfare in low- and middle-income nations provide some insight into the prevalence of adversity in these ecologies.

According to Heleski and colleagues [58], in 2009, of the more than 55 million horses, close to 84% were used as work animals in low-income and developing nations, a pattern that extended to other equids such as donkeys (98% of 41 million) and mules (96 of 13 million). Moreover, according to Heleski and collaborators [58], only 10% of the global equid population is treated by veterinarians specializing in equine care. These cross-national differences in horses’ welfare provide preliminary insight into the nature and quality of horse–human relationships, wherein, due to local ecological challenges, owners are unable or unwilling to provide adequate care for their animals. Bonsi and collaborators [59] performed a systematic review and meta-analysis to determine the influence of socioeconomic factors on the prevalence and severity of diseases in equids used in work-related activities in developing nations. Their study revealed that equids were predominantly affected by epizootic lymphangitis, African horse sickness, equine trypanosomiasis, equine infectious anemia, gastrointestinal helminthiasis, foot diseases, and lameness. Bonsi and colleagues [59] also concluded that the effects of these diseases on diagnosed animals ranged from a reduction in the animals’ work efficiency; a partial or total inability to work; and, in several cases, a higher likelihood of death. In turn, the animals’ illness, injury, or death also impacted the owners’ financial outcomes, such as a reduction in income, unemployment, increased poverty, bill payment, and default due to veterinary treatments [59]. Therefore, these results suggest that environmental adversity has a significant and negative influence on the welfare of horses, as evidenced by a higher risk of suffering infection, injury, or death. In turn, the affliction or death of work equids has a negative impact on the socioeconomic prospects of owners and their families, maintaining or increasing the general level of adversity.

Recent studies have linked horses’ general health and human attitudes towards horses. For example, Haddy and colleagues [60] asked participants about their horse welfare as well as their attitudes and beliefs about animal sentience across six nations: Spain, Portugal, Pakistan, Senegal, Mexico, and Egypt. According to Haddy and collaborators [60], horses had better general health scores when their owners believed that their animals were capable of experiencing emotions compared to participants who held a different view. While this difference varied across countries; remained statistically significant in Senegal, Spain, Portugal, and Egypt, and it disappeared in Mexico [60]. The authors also identified a significant difference in general horse health between caretakers who established an affective relationship with the animal and those who had an instrumental relationship with horses. This effect varied across nations. Whereas it remained statistically significant in Egypt and Senegal, it disappeared in Mexico, Pakistan, Spain, and Portugal [60]. The authors also discovered that a greater number of lame horses were owned by participants who believed that their animals were incapable of experiencing pain. Although future studies are required to determine the contributing factors to the latter cross-national differences, the aforementioned results provide further insight into the positive association between the quality of the human–horse relationship and the general health of equids across nations.

Moreover, considerable differences exist in the production and consumption of horse meat at the cross-national level. According to the Alberta Horse Welfare Report [61], China consumes more horse meat per year (more than 420,000 tons) compared to other nations. Mexico, Russia, Italy, and Kazakhstan also consumed horse meat (ranging between 84,170 and 54,460 tons), albeit not to the same extent as China. Lastly, countries such as Argentina, Mongolia, France, Australia, Kirgizstan, Ukraine, Japan, and Belgium consumed between 9470 and 26,370 tons of horse meat [61]. The Alberta Horse Welfare Report [61] also described that the leading importers of horse meat included Switzerland, Canada, Mexico, Japan, the United States, the Netherlands, Russia, Belgium, and France. Further, the report identified the principal horse meat exporters, such as Uruguay, Mongolia, the Netherlands, France, Canada, Brazil, Poland, Belgium, the United States, and Argentina. Although horse meat continues to be produced, distributed, and consumed, these cross-national data should not be interpreted as evidence supporting the notion that most horses’ behaviors are due to their prey status; as a counter-example, consider the production and consumption of dog meat around the globe. Several nations have, or until recently had, institutions that enabled their citizens to slaughter, sell, and consume canine meat [62,63]; it would be misleading to extrapolate and interpret dogs’ behaviors and the diversity of their relationships with people based on the dietary preferences of specific nations. The same reasoning applies to the consumption of horse meat and the prey hypothesis. Additional studies are required to determine if horses raised for slaughter differ in their cognition and behavior towards humans compared to equids used for non-dietary purposes (work, riding, or companionship).

## 2. Discussion

The prey hypothesis remains a predominant perspective among equestrian trainers and researchers. However, our review of the corresponding literature describing this approach suggests a fundamental lack of empirical evidence supporting the prey hypothesis. Although traditional statistical models revealed a significant effect of diet on orbit orientation (convergence and frontation), subsequent phylogenetic comparative analyses failed to replicate these results, demonstrating that the influence of diet was a product of pseudoreplication, wherein the models’ residuals were not statistically independent. A similar pattern emerged when considering the contribution of foraging mode on species’ pupil orientation, with classical statistical analyses revealing an effect that failed to replicate after accounting for the data’s underlying phylogenetic structure. Consequently, these empirical examinations falsify the following predictions: (1) prey species have lateral orbit orientation; (2) predator species have frontal orbit orientation; and (3) prey species have horizontal pupils. Moreover, as described in this paper, the experimental evidence suggests horses feature accurate stereoscopic vision, falsifying another prediction under the prey hypothesis.

In contrast to the prey hypothesis, the mutualistic coevolution hypothesis argues that horses are cognitively capable of distinguishing between types of threats. As evidenced by the experimental data, horses can indeed discriminate among predators. The prey hypothesis does not predict a contribution of horses’ ontogenetic changes, attachment towards humans, or positive/negative experiences with humans impacting their socio-behavioral repertoire and cognition. In contrast, the mutualistic coevolution hypothesis focuses on the latter predictions. The present review identified empirical evidence supporting the role of ontogeny and positive social experiences with people on horses’ performance in social cognitive tasks.

The prey hypothesis often appears in the literature as an ad hoc explanation concerning a horse’s fearful behavior without considering the source of such behavioral reaction, which could be attributable to sex, age, breed, temperament, or attachment styles. Moreover, the comparative evidence also counters the assertion that so-called prey species (e.g., ungulates) will have greater levels of predator vigilance as a function of group size and have a lateral orbit orientation compared to predator species (carnivorans or humans). As described in this review, diet does not adequately predict these morphological and behavioral features. Additionally, predators are not exempt from being the subject of predation or lethal aggression perpetrated by conspecifics or heterospecifics. The positive correlation between capture myopathy and running speed (a proxy for predation risk) also provides additional insight in that the presence of this physiological condition in several wild equids or its absence in domestic horses confirms that the surviving populations of ancestral horses in the Pontic steppe faced a lower diversity of predators compared to most equids in the Afrotropics [41].

After reviewing the ethological evidence on social cognition in horses, a critic may argue that horses are attuned to human expressions and gestures as they have been subjected to intense predation pressures, requiring the animal to assess the predator’s behavior and potential intentions accurately. However, such a claim ignores the phenotypic overlap in social cognitive abilities between horses and domestic dogs, such as gaze following, understanding pointing, and differentiating between human emotions across sensorial and perceptual modalities. Although additional studies are required to replicate the findings of studies on horses’ reactions to the Strange Situation, preliminary examinations do suggest that entering a novel environment and interacting with a human stranger increases horses’ aversive responses. Horses are not the only species to exhibit distress when confronted with these experimental conditions, as domestic dogs also feature physiological and behavioral features of distress during the early stages of the Strange Situation. In our view, it is more reasonable to think that horses’ avoidance and fearfulness when removed from their herd or familiar location is not because they are a prey species but a social species.

Additional comparative studies are required to determine whether these cognitive abilities are also present in wild equids exposed to high predation risk. It is worth noting that the present paper does not claim that horses lack behaviors or cognitive abilities that reduce the risk of predation. As previously mentioned, horses are capable of distinguishing between different predator species and exhibit an assortment of behavioral responses. However, our analyses call into question the long-asserted view that horse training aims to overcome a horse’s fearfulness due to its prey nature. Instead, we recommend a more nuanced perspective wherein the symbiotic mutualist coevolution between horses and humans for the last 4000 years may have selected for domestic horses’ cognitive, behavioral, affective, and personality traits.

## 3. Conclusions

As shown by the evidence we have reviewed in this paper, the current comparative data does not support the claim that the predator–prey distinction adequately captures the phenotypic variance associated with animals’ cognition and behavior. This is not to deny that animals have morphological, physiological, behavioral, and cognitive adaptions associated with a particular trophic guild. However, to claim that a vast array of a species’ traits are attributable to its diet discounts the influence of additional ecological factors, as is the case with symbiotic associations between horses and humans. Unfortunately, the prey hypothesis ignores the thousands of years of human–horse coevolution and instead perpetuates the outdated notion that horse and human interactions have remained relatively unaltered since the Paleolithic era. Our argument should not be confused with denying the presence of anti-predator strategies in horses; however, most horses’ behaviors should not be interpreted as the expected response of a “prey” species before conducting the pertinent ethological-empirical examination. Following our review of the literature on this subject, we conclude that in most instances where horses are referred to as prey animals, no empirical evidence was provided to justify such an assertion. Several equitation professionals and researchers have also suggested replacing current training procedures based on overcoming horses’ alleged “natural prey distrust” with ones that build on the aforementioned mutualistic inclinations between horses and trainers. Such approaches emphasize developing a secure bond that acknowledges the animal’s socio-cognitive abilities and emotional needs and that reflects the mutualist symbiosis between horses and humans.

## Figures and Tables

**Table 1:** Predictions associated with the prey and mutualistic coevolution hypothesis.

*Predictions*	*Prey* *Hypothesis*	*Mutualistic* *Coevolution Hypothesis*
***Morphology***		
*Prey species have lateral orbit orientation.*	+	None
*Predator species, frontal orbit orientation.*	+	None
*Prey species have horizontal pupils.*	+	None
***Predation***		
*In part, horses’ fear of humans originates from human predation in the ancient past.*	+	None
*Horses distinguish among predators.*	None	+
***Attachment, learning, and quality of interactions with humans***		
*Horses’ attachment styles generalize or extend to their relationship with people.*	None	+
*In addition to ancestral conditions, horses’ social behaviors are attributable to learning and developmental changes.*	None	+
*Positive social experiences with people, in conjunction with learning processes, influence horses’ social cognition.*	None	+
*Adverse captive environments increase the likelihood of behavioral disorders.*	None	+

**Note**. None: indicates that no prediction is associated with the hypothesis; *+*: a positive effect is predicted.

## Data Availability

Data and R code will be made available upon request to the corresponding author.

## Supplementary Materials

The following supporting information can be downloaded at https://www.mdpi.com/article/10.3390/ani15050641/s1. Reference [64] is cited in the Supplementary file.
