# Supplementary material for: Nay to Prey: Challenging the View of Horses as a “Prey” Species
Source: Animals (Basel). 2025 Feb 22;15(5):641. doi: 10.3390/ani15050641 (PMC11898186; doi:10.3390/ani15050641)
Supplement: Supplementary file 1 [file animals-15-00641-s001.zip › animals-3417651-supplementary.pdf]

Supplementary Material

We computed phylogenetic comparative models examining the influence of activity pattern, substrate use, and diet on orbit convergence and frontation in a sample of 194 taxa of mammals, including Artiodactyla, Perissodactyla, Carnivora, and Primates. The analyses revealed sizeable phylogenetic signals for orbit convergence (λ=.976) and orbit frontation (λ=.943), suggesting these morphological traits are phylogenetically conserved. The Phylogenetic Generalized Least Squares Model for orbit convergence did not reach statistical significance (p=.2705) and explained 1.9% of the variance. Consequently, no further interpretations were conducted. In contrast, the Phylogenetic Generalized Least Squares Model for orbit frontation did reach statistical significance (p=.0343) but explained 4.74% of the variance. Neither the activity pattern (β=.017, p=.7889) nor the diet indices (β=-.088, p=.2139) significantly predicted the species' orbit frontation. The substrate index was statistically significant; however, its effect was small in magnitude (β=-.161, p=.0308).

Similarly, we conducted multiple phylogenetic analyses examining the influence of diel activity and foraging mode on pupil orientation in a sample of 132 mammals. The comparative examination of the pupil orientation index suggested a high value for Pagel's λ (1.00), confirming that this trait is conserved across the phylogenetic tree. The analysis did reach statistical significance (p=.0485), which explained 8.25% of the variance. Although the category of nocturnal was significantly different from diurnal (unstandardized b= -.318, p= .0212), polyphasic had no significant effect (unstandardized b= -.244, p= .0573). Foraging mode (ambush: unstandardized b= -1.155, p= .2001; active: unstandardized b= -1.037, p= .2495) had no significant influence on the pupil orientation index.

As a robustness test, subsequent models dichotomized the pupil data into horizontal (0) relative to other pupil orientations (1) and re-examined the effect of diel activity and foraging mode. A Phylogenetic Logistic Regression (using the *phylolm* package [64]) suggested that neither diel activity (nocturnal: estimate= -.001, p=.9959; polyphasic: estimate= -.001, p=.9995) nor foraging mode (ambush: estimate= .000, p=.9999; active: estimate= .000, p=.9998) significantly predicted the species' pupil orientation. These results strongly indicate that foraging mode does not influence pupil orientation after controlling for underlying phylogenetic effects, further questioning the assertion that "prey" species vary in their ocular morphology relative to "predator" taxa.

Lastly, we performed several phylogenetic comparative models examining the influence of diet on orbit convergence and frontation in a sample of nonhuman primates. The phylogenetic signal was particularly high for both convergence and frontation (respectively λ=.979; λ= .788), supporting the value of phylogenetic comparative analyses in revealing the contribution of phylogenetic inertia to ocular orientation. Our first phylogenetic comparative model, including convergence as a criterion variable, reached statistical significance and explained 46.4% of the variance, with omnivores featuring lower convergence (i.e., less binocular vision) relative to frugivores/folivores (β=-.678, p<.0001), whereas frugivores and folivores featuring similar convergence (β=-.039, p=.669). A similar pattern emerged for frontation, with the model reaching statistical significance and explaining 21.2% of the variance: Onmivores displayed lower frontation values (i.e., eyes oriented at an upward/downward angle) compared to frugivores/folivores (β=-.460, p<.0001). In contrast, frugivores and folivores did not differ in frontation scores (β=-.013, p=.906).
